# Post-Acute Sequelae of COVID-19 (PASC) in Hospitalized and Ambulatory Patients: A Comparative Study

**DOI:** 10.3390/jcm15103681

**Published:** 2026-05-11

**Authors:** Magdalena Król-Kulikowska, Marta Kepinska, Justyna Siwy, Felix Keller, Harald Mischak, Ralph Wendt, Emelie Sarenmalm, Åsa Nilsson, Björn Peters, Emmanuel Dudoignon, Fatima Zunara, Morgane Michel, Mercedes Salgueira, Goce Spasovski, Christian Scholz, Patryk Wawrzonkowski, Mirosław Banasik, UriCoV Working Group

**Affiliations:** 1 Department of Pharmaceutical Biochemistry, Faculty of Pharmacy, Wroclaw Medical University, Borowska 211a, 50-556 Wrocław, Poland; marta.kepinska@umw.edu.pl; 2 Mosaiques Diagnostics GmbH, 30659 Hannover, Germany; 3 Department of Internal Medicine IV (Nephrology and Hypertension), Medical University of Innsbruck, 6020 Innsbruck, Austria; 4 Division of Nephrology, St. Georg Hospital, 04129 Leipzig, Germany; ralph.wendt@sanktgeorg.de; 5 Department of Infectious Diseases, Skaraborg Hospital, Region Västra Götaland, 541 85 Skövde, Sweden; 6 Research, Education, Development, and Innovation Department, Skaraborg Hospital, 541 85 Skövde, Sweden; 7 Department of Nephrology, Skaraborg Hospital, Region Västra Götaland, 541 85 Skövde, Sweden; bjorn.peters@vgregion.se; 8 Department of Molecular and Clinical Medicine, Institute of Medicine, The Sahlgrenska Academy at University of Gothenburg, 413 45 Gothenburg, Sweden; 9 Department of Anaesthesiology and Critical Care, Hôpital Saint Louis-Lariboisière, AP-HP, Université Paris Cité, INSERM UMR-S 942 MASCOT, 75010 Paris, France; 10 Université Paris Cité, ECEVE, UMR 1123, Inserm, 75010 Paris, France; 11 Assistance Publique-Hôpitaux de Paris, Hôpital Robert Debré, Service de Santé Publique, Équipe REPERES, 75019 Paris, France; 12 Virgen Macarena Hospital, University of Seville, 41009 Seville, Spain; 13 Department of Nephrology, University Sts. Cyril and Methodius, 1000 Skopje, North Macedonia; 14 University Hospital Cologne, Internal Medicine II-QiN-Quality in Nephrology, 50935 Köln, Germany; 15 KfH Kuratorium Für Dialyse und Nierentransplantation e.V., Medical Division, 63263 Neu-Isenburg, Germany; 16 Department of Nephrology, Transplantation Medicine and Internal Diseases, Institute of Internal Diseases, Wroclaw Medical University, 50-556 Wrocław, Poland

**Keywords:** ambulatory, COVID-19, hospitalized, long COVID, PASC

## Abstract

**Background/Objectives**: COVID-19 may result in persistent symptoms, some of which can be disabling, referred to as post-acute sequelae of SARS-CoV-2 (PASC, or long COVID). The growing recognition of PASC as a public health challenge underscores the need for comprehensive studies of its course, risk factors, and clinical outcomes. This multicentric study aimed to compare the prevalence, symptom spectrum, and functional impact of PASC in two patient groups: those hospitalized with COVID-19 and those managed exclusively in ambulatory care. **Methods**: Molecular, clinical, and demographic data were obtained from 319 patients diagnosed with COVID-19 in seven European countries. Patients were classified as PASC in accordance with the WHO definition. **Results**: PASC is more frequent and severe among hospitalized patients, while ambulatory patients present with milder but prolonged symptoms. Categorical age analysis suggests that older age was associated with a higher incidence of PASC, highlighting the need for multidisciplinary management and targeted support. **Conclusions**: Our findings highlight distinct differences in the presentation and impact of PASC across patient groups, with important implications for individualized care, multidisciplinary management, and effective healthcare planning in the post-pandemic era.

## 1. Introduction

The coronavirus disease 2019 (COVID-19), caused by the severe acute respiratory syndrome coronavirus 2 (SARS-CoV-2), has posed unprecedented challenges to global health systems, economies, and societies since its identification in Wuhan, China, in December 2019 [1,2]. By disrupting nearly every aspect of daily life, the pandemic has led to more than 779 million confirmed cases and over 7.1 million deaths globally as of early 2026 [3]. The initial medical focus centered on acute respiratory manifestations, hospitalizations, and mortality; however, as the pandemic evolved, it became increasingly apparent that a subset of individuals continued to experience a diverse array of symptoms well beyond the resolution of the acute phase of infection [4,5,6]. This clinical entity has been recognized as post-acute sequelae of SARS-CoV-2 (PASC), more commonly referred to as long COVID [4]. According to current definitions, PASC refers to symptoms that persist for at least four weeks after the initial infection, although some frameworks, including those by the U.S. Centers for Disease Control and Prevention (CDC), classify symptoms lasting four to twelve weeks as part of the chronic phase [7,8].

PASC encompasses a wide range of symptoms, including fatigue, dyspnea, post-exertional malaise, cognitive dysfunction, myalgia, sleep disturbances, chest pain, palpitations, anosmia, gastrointestinal symptoms, and neuropsychiatric issues such as anxiety and depression [9,10,11]. These symptoms can persist for weeks or months and may significantly impair daily functioning and quality of life [11].

The pathophysiological mechanisms behind PASC remain incompletely understood, with hypotheses including viral persistence, immune dysregulation, autoimmunity, microvascular injury, and post-critical illness sequelae [12].

Importantly, PASC can affect individuals across the entire spectrum of initial disease severity—from those requiring intensive care unit admission to those with initially mild or even asymptomatic infections [13,14]. This highlights the necessity of a broader public health response beyond the acute care setting, especially as millions of people worldwide have recovered from the acute phase but may face ongoing health issues impacting their quality of life, functional status, and capacity to return to work or education [15].

Reported prevalence rates of PASC vary widely, ranging from approximately 10% to over 50%, depending on study design, population demographics, diagnostic criteria, and follow-up intervals [16,17]. Some studies suggest that women, individuals with pre-existing comorbidities, and those who experienced more severe acute disease may be at increased risk, though findings remain inconsistent [18]. However, direct comparisons between hospitalized and non-hospitalized patients in terms of symptom burden, duration, and functional outcomes remain limited. Identifying and understanding the risk factors and clinical course of PASC in different patient groups is essential for developing appropriate follow-up strategies and healthcare resource planning [19]. In this context, our study aims to compare the prevalence, spectrum, and functional impact of PASC symptoms in two distinct patient groups: individuals previously hospitalized with COVID-19 and those managed entirely in the outpatient setting. By integrating patient-reported outcome measures, we seek to identify unique symptom trajectories and potential determinants of prolonged recovery. Moreover, this study also attempted to identify risk factors that may influence the development of PASC in patients. Understanding these differences will not only inform individualized patient management but also guide health system planning and resource allocation in the post-pandemic era.

## 2. Materials and Methods

### 2.1. Study Population 

A prospective cohort study was conducted to identify baseline characteristics associated with later PASC and to evaluate potential predictors of PASC development. Among the 1012 COVID-19 patients included in the “Prospective Validation of a Proteomic Urine Test for Early and Accurate Prognosis of Critical Course Complications in Patients with SARS-CoV-2 Infection” (CRIT-COV-U; study registered in the German Clinical Trials Register, study number: DRKS00022495) study, 769 survived the acute phase of the disease and were re-contacted. Of these, 319 patients completed and returned the questionnaire, and this group was included in further analyses. The questionnaire was developed in 2023 and translated by healthcare professionals into six languages, then sent to the participants’ homes. Contact information was obtained from the electronic hospital registry. Alternatively, participants were contacted by phone and invited to complete a telephone-based survey conducted by medical research assistants. Participants were enrolled after completing a positive questionnaire between January 2023 and July 2024. This manuscript is a clinical sub-analysis of the results of the research and responses of patients who were initially recruited as part of the CRIT-COV-U project.

Molecular, clinical, and demographic data were collected from 319 patients with COVID-19 as part of the multicenter “URInary Peptidomic Patterns of Long-COVID Syndrome” (UriCoV) study, which was a continuation of the CRIT-COV-U study [20]. Characteristics of patients by country are presented in Table 1. This project complied with the Declaration of Helsinki. The Bioethics Committee of the Wroclaw Medical University (Wroclaw, Poland; number 475/2024) and the Institutional Review Boards of the recruitment centers granted ethical approval.

The questionnaire was developed by the UriCoV consortium, based on Delphi consensus criteria consistent with the World Health Organization (WHO) definition [8]. The questionnaire was designed to identify individuals with suspected post-COVID or PASC condition (Supplementary Materials). The identification was based on participants’ responses to the first four items of the questionnaire, which assessed (1) whether symptoms or complaints persisted since the initial acute COVID-19 infection; (2) whether new complaints emerged within three months following the first acute COVID-19 infection; (3) whether any symptoms or complaints resulted in new health limitations; and (4) whether a pre-existing underlying condition worsened since the initial COVID-19 infection and the symptom duration. Individuals classified as PASC answered at least 1 of the 4 questions with a “yes” and had symptoms for at least 2 months. The criteria of the questionnaire and the design of the study are described in detail in another article [21].

The patients were recruited from seven countries (Poland, Austria, Germany, Spain, France, North Macedonia, Sweden), with the largest number of respondents coming from North Macedonia (*N* = 92), Germany (*N* = 82), and Poland (*N* = 76). In North Macedonia and Poland, the majority of patients were ambulatory, whereas in the remaining five countries, hospitalized patients were included in the study almost exclusively. Variables such as inclusion status, WHO score, estimated glomerular filtration rate (eGFR), systolic and diastolic pressure, smoking status, and the presence of chronic diseases were obtained from the CRIT-COV-U study. Other data (duration of illness, number of days missed from work) were obtained from questionnaires.

The demographic data and clinical characteristics of patients are presented in Table 1.

### 2.2. Statistical Analysis

The questionnaire was anonymous. It was manually coded and stored in the REDCap database. Statistical analyses were performed using the STATISTICA 13.3 software package (Statsoft Polska, Sp. z o.o., Krakow, Poland), licensed to Wroclaw Medical University. The normality of continuous variables was assessed using the Shapiro–Wilk test, and the homogeneity of variance was tested using Levene’s test.

To test the hypotheses about the existing differences between the study groups, the Mann–Whitney U test for two groups of continuous variables, the Kruskal–Wallis test for more than two groups of continuous variables, and Pearson’s χ^2^ test for categorical variables were used. For small-sized groups, Yates’s correction for continuity was applied.

A logistic regression was performed to assess associations between selected parameters and the risk of developing PASC. The results are presented as odds ratios (OR) with 95% confidence intervals (CI). Statistical significance was defined at *p* < 0.05.

## 3. Results

### 3.1. The Comparison of Non-PASC and PASC Patients

Analyzing patients with and without PASC, no statistically significant differences were found in the following contexts: sex (*p* = 0.592), smoking status (*p* = 0.141), systolic blood pressure (*p* = 0.391), diastolic blood pressure (*p* = 0.715), and history of cancer (*p* = 0.532).

PASC patients were statistically older than non-PASC patients (*p* = 0.001), displaying higher BMI (*p* = 0.011), and lower eGFR (*p* = 0.011). Furthermore, PASC patients had a higher percentage of hospitalizations than non-PASC patients (*p* < 0.001). In addition, these patients were more likely to report chronic diseases such as diabetes (*p* = 0.005), hypertension (*p* = 0.001), heart failure (*p* = 0.048), and heart disease (*p* = 0.004). PASC patients also reported more work days missed (*p* = 0.001), as well as a longer duration of illness (*p* < 0.001).

The results are presented in Table 2.

### 3.2. The Comparison of Ambulatory and Hospitalized Patients

When examining the two groups—ambulatory and hospitalized patients—no statistical difference was found in terms of BMI (*p* = 0.185), diastolic blood pressure (*p* = 0.581), and the duration of the disease (*p* = 0.446).

A statistically significant difference was found in the context of the patients’ country of origin (*p* < 0.001). The data from North Macedonia and Poland showed a clear predominance of ambulatory patients. In turn, PASC was observed more often in hospitalized patients compared to ambulatory patients (*p* < 0.001). PASC was diagnosed in 56% of hospitalized patients, while it was detected in only 27% of ambulatory patients. A difference in age was also observed. Hospitalized patients were statistically older compared to ambulatory patients (*p* < 0.001). A difference was also observed in the sex distribution between the ambulatory and hospitalized groups (*p* = 0.004). In the former, a predominance of men was observed (56%), while in the latter, a predominance of women was observed (61%). It was also shown that baseline eGFR was lower (*p* < 0.001) and baseline systolic blood pressure was higher (*p* < 0.001) in hospitalized patients compared to ambulatory patients.

Moreover, statistically significant differences between the study groups were observed for smoking status (*p* < 0.001), presence of diabetes (*p* < 0.001), history of hypertension (*p* < 0.001), history of heart failure (*p* = 0.006), history of heart disease (*p* < 0.001), and history of cancer (*p* = 0.021). Ambulatory patients were more likely to smoke cigarettes, but hospitalized patients were more likely to have chronic diseases. It was also observed that hospitalized patients missed work more often compared to ambulatory patients (*p* < 0.001).

The results described above are presented in Table 3.

### 3.3. The Comparison of Patients Divided by Age

After dividing patients into five groups based on age (20–39 years, 40–49 years, 50–58 years, 59–68 years, 69–90 years), no statistically significant differences were observed between these groups in terms of sex distribution (*p* = 0.846), diastolic blood pressure (*p* = 0.377), and history of cancer (*p* = 0.152). The results in most age groups in the context of country, ethnicity, and the scores on the WHO scale were too low to be compared with each other.

However, a relationship was found between age group and the occurrence of PASC (*p* = 0.016). In patients aged 49 years and older, the percentage of PASC fluctuated around 50%. There was also a correlation between belonging to a specific age group and the BMI (*p* = 0.025). Moreover, statistically significant differences were also observed between the groups in terms of eGFR (*p* < 0.001), systolic blood pressure (*p* = 0.004), smoking status (*p* < 0.001), presence of diabetes (*p* < 0.001), history of hypertension (*p* < 0.001), history of heart failure (*p* < 0.001), and history of heart disease (*p* < 0.001).

A difference was also observed between a specific age group and patient status at inclusion (*p* < 0.001). The older the age, the higher the percentage of hospitalized patients. In the oldest group, this percentage reached 100%. Moreover, a relationship was found between age and the duration of PASC (*p* = 0.030). Patients from older groups were ill longer compared to patients from younger groups. This was probably related to the number of work days missed, as patients from older groups missed more days compared to younger patients (*p* = 0.017).

The results are presented in Table 4.

### 3.4. The Comparison of Hospitalized and Ambulatory Patients in Terms of the Frequency of Consulting Selected Specialists

There was no difference between the status at inclusion (hospitalized patients, ambulatory patients) and cardiologist (*p* = 0.609), neurologist (*p* = 0.391), psychiatrist (*p* = 0.395), and general practitioner (*p* = 0.751) consultations. The results regarding the need to consult a pneumologist and a physiotherapist were on the borderline of statistical significance (*p* = 0.057 and *p* = 0.065, respectively).

The results are summarized in Table 5.

### 3.5. The Comparison of PASC and Non-PASC Patients in Terms of the Frequency of Consulting Selected Specialists

There was no association between the occurrence of PASC and the frequency of pneumologist (*p* = 0.308), neurologist (*p* = 0.415), psychiatrist (*p* = 0.712), physiotherapist (*p* = 0.214), and general practitioner (*p* = 0.706) consultations.

However, it was noted that patients with PASC more frequently reported the need for cardiology consultation compared to patients who did not develop PASC (*p* = 0.017).

The results described above are presented in Table 6.

### 3.6. The Comparison of Patients by Anxiety and Depression Levels

There was no relationship between the level of anxiety and depression and sex distribution, although the result was on the borderline of statistical significance (*p* = 0.067).

However, there was a relationship between the level of anxiety and depression and the occurrence of PASC (*p* < 0.001). The higher the level of anxiety or depression, the greater the percentage of patients diagnosed with PASC. A similar relationship was found for the status at inclusion (*p* = 0.046). The higher the level of anxiety or depression, the greater the percentage of hospitalized patients.

The results described above are summarized in Table 7.

### 3.7. The Influence of Various Factors (Age, Sex, Status at Inclusion) on the Risk of Developing PASC

In this study, logistic regression analysis was performed to evaluate the influence of the following factors: age, sex, BMI, eGFR, systolic blood pressure, status at inclusion, presence of diabetes, history of hypertension, history of heart failure, and history of heart disease on the risk of developing PASC. No significant associations were found between most parameters and the risk of disease. These parameters included country (Austria, *p* = 0.577; Germany, *p* = 0.685; France, *p* = 0.805; North Macedonia, *p* = 0.726; Sweden, *p* = 0.601), age (*p* = 0.949), sex (*p* = 0.150), eGFR (*p* = 0.457), systolic blood pressure (*p* = 0.474), smoking status (*p* = 0.498), presence of diabetes (*p* = 0.811), history of hypertension (*p* = 0.260), history of heart failure (*p* = 0.369), and history of heart disease (*p* = 0.115). The relationship between the risk of PASC and BMI (*p* = 0.085) was on the borderline of statistical significance.

However, it was observed that hospitalized patients had more than 3 times higher risk of developing PASC compared to ambulatory patients (*p* < 0.001). In the case of Spain, a lower probability of PASC occurrence was observed compared to Poland (*p* = 0.042), but this result should be interpreted with caution due to the small sample size. The findings are presented in Table 8.

## 4. Discussion

This multicenter study provides important insights into the epidemiological and clinical characteristics of patients with PASC across different European countries. By comparing data from Poland and several other European nations (Austria, Germany, Spain, France, North Macedonia, and Sweden), we observed significant differences in age distribution and the prevalence of PASC. These differences suggest both biological and systemic healthcare factors contribute to PASC risk and management.

This is important because PASC is a clinical unit that covers many organ systems, which poses a serious challenge for our society and the healthcare system. In addition to the symptoms assessed in this study, a wide range of complications were described, including thrombotic events [22], myocarditis [23], or postural orthostatic tachycardia syndrome (POTS) [24]. Respiratory system involvement may also include diaphragm dysfunction [25], and increasingly recognized are neurological complications, such as cognitive impairment and peripheral neuropathies [26]. These observations emphasize the complex and multifactorial nature of PASC and should be considered when interpreting our findings.

### 4.1. Geographical Differences and Age and Sex-Related Trends

Our data indicate an apparent lower incidence of hospitalization and PASC among patients from Poland and North Macedonia compared to those from other European countries in this study. This is most likely due to the fact that patients from Poland and North Macedonia generally were recruited in ambulatory settings due to a less severe disease course, while in the other countries, patients were recruited in clinics, experiencing more severe COVID-19.

The lower rate of PASC diagnosis in Poland and North Macedonia in this study may also result from underdiagnosis due to less systematic screening for post-acute sequelae, as highlighted by Fernández-de-las-Peñas et al. [27], who stressed the importance of active follow-up and standardized definitions in PASC studies.

Older patients, particularly those over 65, were more likely to experience PASC symptoms. This is consistent with existing literature indicating that age is one of the most robust predictors of persistent post-COVID symptoms [16]. Older adults may have a reduced physiological reserve, a higher prevalence of comorbidities, and be more susceptible to the long-term effects of systemic inflammation.

In terms of sex, our results suggest that men were more frequently hospitalized, yet women were slightly more represented among PASC patients—a pattern observed in other cohorts [28]. This paradox may be explained by the fact that while men are at higher risk for severe acute COVID-19, women are more likely to report persistent symptoms, possibly due to differences in immune responses and health-seeking behavior. The observed age distribution differences between countries (e.g., older German patients vs. younger North Macedonian patients) may reflect demographic realities but may also be due to selection bias introduced as a result of ambulatory vs. clinic recruitment, also influencing PASC incidence. As age is a major risk factor, populations with younger cohorts may naturally report fewer PASC cases. However, differences in variant circulation (e.g., Delta vs. Omicron), vaccination rates, and social behaviors may also be significant [29,30].

### 4.2. Hospitalization and PASC

Our study revealed a strong and statistically significant association between prior hospitalization due to acute COVID-19 and the subsequent development of PASC symptoms. This relationship is consistent with numerous international studies, suggesting that the severity of the initial infection is a major risk factor for prolonged post-viral symptoms [11,31]. Patients who required hospitalization, especially those admitted to intensive care units (ICUs), were significantly more likely to report fatigue, dyspnea, cognitive difficulties, and musculoskeletal pain weeks to months after discharge. Al-Aly et al. [31], using data from the U.S. Veterans Health Administration, found that individuals who had been hospitalized for COVID-19 had markedly elevated risks for nearly all PASC symptoms across multiple organ systems, even when compared to matched non-COVID controls. These risks were highest among those who required mechanical ventilation, reinforcing the idea that disease severity plays a central role in long-term outcomes. One potential mechanism underlying this association is sustained systemic inflammation. Severe COVID-19 has been characterized by a dysregulated immune response and a pro-inflammatory cytokine storm [32], which may lead to long-lasting damage to tissues and organs, including the lungs, heart, brain, and kidneys. In addition, prolonged immobility during hospitalization can contribute to deconditioning, muscle atrophy, and venous thromboembolism, all of which may contribute to the persistence of symptoms such as fatigue and dyspnea. Another important factor is the psychological impact of hospitalization itself. Prolonged ICU stays are known to be associated with post-intensive care syndrome (PICS), which includes cognitive impairment, anxiety, depression, and post-traumatic stress disorder (PTSD). These neuropsychiatric symptoms are commonly reported in PASC cohorts and may be partly attributable to the psychological trauma of hospitalization and the experience of severe illness [33].

Interestingly, our data also showed that patients who were hospitalized during their acute infection reported missing significantly more days of work or study compared to non-hospitalized individuals. These findings are, of course, relevant to acute COVID-19, but exploring how PASC translates into these data would be extremely useful. This would confirm the substantial socioeconomic burden of PASC noted in recent European cohort studies [34]. This would also be consistent with findings from the REACT-Long COVID study in the United Kingdom [35], which reported greater functional impairment and lower return-to-work rates among previously hospitalized COVID-19 patients. The economic implications of this are substantial and highlight the need for structured return-to-work and rehabilitation programs targeting this vulnerable subgroup. It is worth noting that while hospitalization is a clear risk factor, PASC is not limited to patients with severe disease. Some individuals with mild or even asymptomatic infections also develop persistent symptoms, suggesting that other mechanisms—such as viral persistence, autoimmunity, or dysautonomia—may also play a role [36]. However, the burden and complexity of symptoms appear greater among those who were hospitalized, reinforcing the need for risk stratification and prioritization of follow-up care.

### 4.3. Symptoms of PASC

In our cohort, symptoms of PASC were highly heterogeneous, both in nature and duration. The most frequently reported issues included persistent fatigue, shortness of breath, cognitive dysfunction, musculoskeletal pain, and psychological symptoms such as anxiety and depression (the frequency of these symptoms and various medical events in Polish patients is presented in the Supplementary Materials). This clinical picture is consistent with findings from international cohort studies and patient-led research [10,11]. The majority of symptoms were most severe during the first three months post-infection. Still, a substantial proportion of patients (over 30% in our sample) reported complaints for six months or longer. This aligns with findings from the Patient-Led Research Collaborative, where 85% of respondents indicated cyclical recurrence of symptoms [10]. Fatigue emerged as one of the most debilitating and commonly reported PASC symptoms. Often resistant to rest, it significantly impaired individuals’ ability to work, study, and engage in daily activities. Importantly, it did not correlate linearly with the severity of the initial illness. For example, Townsend et al. [37] observed that 52% of patients experienced substantial fatigue after mild acute COVID-19, underlining that PASC is not exclusive to hospitalized cases. Neurocognitive symptoms—especially “brain fog”—included impairment of attention, short-term memory, information processing, and temporal orientation. Some participants also reported language difficulties, disorientation, and cognitive slowing. Recent neuroimaging studies, such as that by Douaud et al. [38], have shown structural brain changes post-COVID, particularly in the orbitofrontal cortex and hippocampus, which may underlie some of these persistent neurological complaints. Pulmonary function testing in follow-up studies has revealed a range of abnormalities, including reduced diffusing capacity for carbon monoxide (DLCO), indicative of impaired alveolar gas exchange [39]. This supports a hypothesis of residual pulmonary inflammation or fibrosis as a contributing factor to long-term respiratory symptoms.

### 4.4. Impact on Daily Life and Work

The functional consequences of PASC can be profound, affecting physical, psychological, and social dimensions of life. One of the most common and disabling symptoms is fatigue, which can be severe enough to interfere with basic daily tasks, including personal care, cooking, or walking short distances [10]. Many patients describe this fatigue as “crash-like” or post-exertional malaise, which is exacerbated by minor physical or cognitive effort—an observation reminiscent of myalgic encephalomyelitis/chronic fatigue syndrome (ME/CFS) [40]. Cognitive impairments—often termed brain fog—are frequently reported and may include problems with memory, attention, word-finding, and executive function [41]. These cognitive difficulties are particularly disruptive for individuals in knowledge-based professions or those pursuing education, often leading to reduced productivity or the inability to return to prior roles. A significant proportion of individuals with PASC report prolonged work absence or reduced work capacity, as our study also demonstrated. In a large international survey, more than 45% of respondents indicated they were working reduced hours or had not returned to work several months after infection [10]. This loss of productivity has implications not only for the individual but also for broader economic and workforce planning, especially in sectors already impacted by labor shortages.

### 4.5. Specialist Consultations and Symptom Clusters

The observed patterns of healthcare utilization in our cohort offer important insights into the clinical burden of PASC. Hospitalized patients—particularly those meeting the criteria for PASC—were more likely to require specialist consultations, most notably in pulmonology, cardiology, and physiotherapy. On the other hand, it should be remembered that hospitalized patients (especially with PASC) usually have more comorbidities, which may be the reason for the increased frequency of consulting specialists. However, results from large-scale cohort studies [10,42,43,44,45] confirm that respiratory and cardiovascular symptoms such as dyspnea, chest pain, and palpitations are among the most persistent manifestations of post-acute COVID-19. Our results further support that hospitalization during acute infection is a strong predictor of more complex post-COVID trajectories. In line with prior research [46,47,48], we found that many previously hospitalized patients continued to experience impaired pulmonary function and physical deconditioning months after discharge, underscoring the need for sustained, multidisciplinary follow-up in this population.

Interestingly, cardiologist consultations were significantly more common among PASC patients even in the ambulatory group, which supports hypotheses about autonomic dysfunction and subclinical myocardial injury in PASC [49]. In light of these findings, future studies should explore the utility of routine cardiac screening, especially in patients reporting persistent fatigue, tachycardia, or exercise intolerance. However, this association may partly reflect the presence of pre-existing cardiovascular disease prior to the COVID-19 pandemic. Notably, one of the most recent studies [50] suggests that after excluding patients with pre-existing symptoms and comorbidities, the proportion of individuals experiencing post-SARS-CoV-2 complications appears substantially smaller than initially estimated.

Psychologically, PASC contributes to increased levels of anxiety, depression, and PTSD, particularly among those who experienced severe acute illness or an ICU stay [51]. These mental health burdens are compounded by the uncertainty of recovery and the lack of clear treatment pathways. Social relationships are also affected. Many patients describe feeling isolated, misunderstood, or stigmatized—especially when their symptoms are invisible or dismissed by healthcare providers or employers [52]. This highlights the urgent need for supportive services, patient advocacy, and improved awareness among clinicians and policymakers. Taken together, the impact of PASC extends well beyond physical health, affecting nearly every aspect of life. Addressing these challenges requires a multidisciplinary approach that includes medical, psychological, social, and occupational rehabilitation strategies.

A striking finding in our analysis was the strong correlation between anxiety, depression, and PASC. Patients with mental health symptoms were more likely to report PASC, regardless of hospitalization status. This supports previous work [17] demonstrating a bidirectional relationship between COVID-19 and mental health disorders. It is still unclear whether psychological distress predisposes individuals to perceive or develop PASC symptoms, or if PASC itself exacerbates underlying or new-onset psychiatric conditions.

This underscores the need for mental health screening and support in all patients with persistent COVID-related symptoms. Multidisciplinary care models, integrating psychologists and psychiatrists alongside primary care and rehabilitation providers, may improve outcomes and reduce chronic disability.

### 4.6. Predictors of PASC

A growing body of literature has aimed to identify predictors of PASC in order to guide clinical follow-up and risk stratification. Several sociodemographic, clinical, and biological factors have emerged as significant predictors. This is not surprising, as many of these factors overlap with the criteria used to define PASC, which potentially strengthens their apparent predictive value [53,54]. Therefore, their role as independent predictors should be interpreted with caution. One of the most consistent findings across studies is the higher prevalence of PASC among women compared to men [11,55]. While the reasons behind this sex difference are not fully understood, hypotheses include hormonal influences, immune system differences, and sociocultural reporting patterns [56]. Although there are indications that sex is important, our study did not demonstrate its influence on the increased risk of developing PASC. Age is another important predictor. Although PASC has been reported across all age groups, older adults appear to be more susceptible to prolonged symptoms, especially those with multiple comorbidities [57]. Nevertheless, younger individuals—particularly those in their 30s and 40s—are not exempt, as several studies report significant rates of persistent symptoms in younger populations, likely reflecting the broader demographics of initial SARS-CoV-2 infection in the community [16]. Although age was not a significant predictor of PASC in the logistic regression model (*p* = 0.949), this likely reflects the limited variability of age in our cohort, where most participants were aged 40–65. Importantly, the analysis of age in a categorical manner revealed a significant association with the frequency of PASC (*p* = 0.016), suggesting a nonlinear relationship. These results suggest that the effect of age on the risk of PASC may not be detectable in models assuming a linear relationship and help reconcile the results of this study with previous studies in which age was a risk factor.

The severity of the acute phase of COVID-19 has also been linked with a higher risk of PASC. Individuals who experienced more symptoms during the initial infection or required hospitalization, particularly ICU admission, are more likely to experience long-term sequelae [9,58,59]. Additionally, high viral load at diagnosis and certain biomarkers, such as elevated D-dimer or IL-6 levels, may correlate with increased risk [60]. Interestingly, pre-existing mental health conditions, such as anxiety or depression, have also been associated with a higher likelihood of PASC, suggesting that psychosocial factors may influence recovery trajectories [17]. This has led to increased interest in the biopsychosocial model when understanding and managing PASC. Despite these findings, predictive models remain imperfect. PASC has been observed even in young, healthy individuals with mild initial infections, highlighting the need for further longitudinal research and biomarker discovery to refine risk assessment tools.

Finally, from a public health standpoint, our findings emphasize the importance of prevention strategies—particularly vaccination—which have been shown to significantly reduce the risk of hospitalization and, consequently, PASC [61]. Reducing the incidence of severe disease through vaccination and early treatment could indirectly mitigate the long-term healthcare burden of post-COVID sequelae.

### 4.7. Limitations

This study has several significant limitations that should be considered when interpreting its results. First, there is a risk of bias resulting from differences in patient recruitment between countries. In some centers, the study enrolled primarily ambulatory patients, while in others, hospitalized patients predominated, which could have impacted the heterogeneity of the study population and the comparability of results.

Second, the study did not include a detailed clinical assessment of PASC. The lack of a standardized, objective clinical assessment of PASC symptoms limits the ability to fully interpret the relationships between reported symptoms and the analyzed parameters and may lead to underestimation or overestimation of the prevalence of this syndrome.

Thirdly, the study did not take into account the influence of the applied therapy due to the heterogeneity of treatment protocols, the time of administration of the drugs, and the relatively limited sample size for individual therapies. Data on vaccination and reinfection were also not taken into account due to the significant heterogeneity of vaccination schemes (different types of vaccines, number of doses, and time in relation to infection). Moreover, the sample size did not allow for subgroup analyses of sufficient power to reliably assess their impact.

Furthermore, despite its prospective cohort design, the observational nature of the study precludes drawing causal conclusions, and the results should be considered descriptive. The study is mainly based on the results reported by patients, which may be prone to errors in recall and reporting; there are no objective clinical or physiological measurements. Future studies would recommend the use of uniform patient selection criteria and the inclusion of a comprehensive clinical assessment of PASC to increase the reliability and comparability of results.

## 5. Conclusions

This study highlights significant differences in the prevalence and profile of post-acute sequelae of COVID-19 (PASC) between hospitalized and ambulatory patients. Hospitalized individuals were more likely to experience persistent symptoms, including fatigue, dyspnea, and cognitive impairment, whereas ambulatory patients more commonly reported mild, yet prolonged, symptoms such as anosmia, headache, or sleep disturbances. Moreover, in patients treated in a hospital, the acute form of COVID-19 lasts longer, and patients are more often absent from work for this reason.

Furthermore, the higher the age, the higher the incidence of patients diagnosed with PASC and hospitalization, which requires greater emphasis on diagnosis and treatment among older adults. Specialist support is also important, as patients with PASC are more likely to require cardiology consultation compared to those without PASC. Our results reinforce the need for a multidisciplinary approach to PASC management, including physical rehabilitation, mental health support, and primary care follow-up, regardless of the initial severity of the disease.

## Figures and Tables

**Table 1:** Characteristics of the study groups divided by country.

Parameter	Overall (*N* = 319)	Country							*p*-Value
		Austria (*N* = 14)	Germany (*N* = 82)	Spain (*N* = 22)	France (*N* = 18)	North Macedonia (*N* = 92)	Poland (*N* = 76)	Sweden (*N* = 15)	
PASC [1 = Yes]	143 (45%)	8 (57%)	48 (59%)	6 (27%)	12 (67%)	28 (30%)	33 (43%)	8 (53%)	0.001
Age [years]	55 (41–65)	50 (42–56)	65 (54–77)	59 (47–63)	62 (49–70)	44 (36–52)	54 (40–65)	61 (51–66)	<0.001
Ethnicity									-
Asian	1 (0.3%)	0 (0%)	0 (0%)	0 (0%)	1 (5.6%)	0 (0%)	0 (0%)	0 (0%)	-
African	1 (0.3%)	0 (0%)	0 (0%)	0 (0%)	1 (5.6%)	0 (0%)	0 (0%)	0 (0%)	-
Hispanic	3 (0.9%)	0 (0%)	0 (0%)	2 (9.1%)	1 (5.6%)	0 (0%)	0 (0%)	0 (0%)	-
Other	5 (1.6%)	2 (14%)	0 (0%)	0 (0%)	3 (17%)	0 (0%)	0 (0%)	0 (0%)	-
Unknown	20 (6.3%)	0 (0%)	0 (0%)	20 (91%)	0 (0%)	0 (0%)	0 (0%)	0 (0%)	-
White	289 (91%)	12 (86%)	82 (100%)	0 (0%)	12 (67%)	92 (100%)	76 (100%)	15 (100%)	-
Sex [1 = Male]	157 (49%)	7 (50%)	34 (41%)	18 (82%)	12 (67%)	45 (49%)	31 (41%)	10 (67%)	0.008
BMI [kg/m^2^]	{24.7; 26.8; 30.3}	{26.9; 31.6; 36.8}	{25.1; 27.0; 30.7}	{25.2; 25.9; 27.7}	{23.2; 26.3; 27.2}	{23.8; 26.5; 30.2}	{24.0; 27.0; 30.6}	{26.0; 27.2; 32.8}	0.058
Smoking Status [1 = Yes]	41 (13%)	2 (14%)	2 (2.4%)	2 (9.1%)	0 (0%)	26 (28%)	9 (12%)	0 (0%)	-
eGFR [mL/min/1.73 m^2^]	99 (88–114)	90 (90–90)	91 (70–110)	104 (93–117)	136 (110–152)	107 (99–115)	92 (88–104)	97 (89–100)	<0.001
Systolic Blood Pressure [mmHg]	124 (115–135)	122 (120–127)	127 (117–138)	119 (115–131)	119 (109–126)	120 (110–127)	129 (120–140)	129 (122–136)	<0.001
Diastolic Blood Pressure [mmHg]	77 (70–83)	73 (69–80)	78 (69–83)	77 (69–85)	68 (62–73)	77 (70–81)	80 (73–88)	75 (69–83)	<0.001
WHO									
1	115 (36%)	0 (0%)	1 (1.2%)	0 (0%)	0 (0%)	74 (80%)	40 (53%)	0 (0%)	-
2	10 (3.1%)	0 (0%)	3 (3.7%)	0 (0%)	0 (0%)	6 (6.5%)	1 (1.3%)	0 (0%)	-
3	46 (14%)	3 (21%)	17 (21%)	1 (4.5%)	0 (0%)	4 (4.3%)	17 (22%)	4 (27%)	-
4	92 (29%)	8 (57%)	40 (49%)	15 (68%)	0 (0%)	8 (8.7%)	15 (20%)	6 (40%)	-
5	40 (13%)	3 (21%)	21 (26%)	6 (27%)	4 (22%)	0 (0%)	2 (2.6%)	4 (27%)	-
6	16 (5.0%)	0 (0%)	0 (0%)	0 (0%)	14 (78%)	0 (0%)	1 (1.3%)	1 (6.7%)	-
Patient Status at Inclusion [1 = Hospitalized]	195 (61%)	14 (100%)	78 (95%)	22 (100%)	18 (100%)	12 (13%)	35 (46%)	15 (100%)	<0.001
Presence of Diabetes [1 = Yes]	53 (17%)	3 (21%)	19 (23%)	3 (14%)	8 (44%)	3 (3.3%)	13 (17%)	4 (27%)	-
History of Hypertension [1 = Yes]	124 (39%)	3 (21%)	46 (56%)	7 (32%)	9 (50%)	25 (27%)	27 (36%)	7 (47%)	0.001
History of Heart Failure [1 = Yes]	16 (5.0%)	0 (0%)	7 (8.5%)	1 (4.5%)	0 (0%)	2 (2.2%)	3 (3.9%)	3 (20%)	0.049
History of Heart Disease [1 = Yes]	23 (7.2%)	1 (7.1%)	11 (13.4%)	1 (4.5%)	0 (0%)	2 (2.2%)	5 (6.6%)	3 (20%)	0.035
History of Cancer [1 = Yes]	15 (4.7%)	0 (0%)	6 (7.3%)	2 (9.1%)	1 (5.6%)	1 (1.1%)	4 (5.3%)	1 (6.7%)	0.703
Duration of Illness [months]	9 (2–9)	6 (1–9)	9 (4–9)	3 (0–9)	4 (3–9)	9 (4–9)	9 (2–9)	9 (9–9)	0.041
Number of Work Days Missed [days]	30 (14–90)	45 (19–60)	78 (35–111)	90 (28–93)	120 (45–365)	-	20 (14–30)	7 (0–150)	1.000

Legend: *N*—number of patients. The “WHO” parameter refers to the scores according to the WHO 8-point clinical progression scale. Values are shown as *N* (%) or median (IQR25–IQR75). The statistical significance of differences was assessed between all study groups.

**Table 2:** Characteristics of non-PASC and PASC patients.

Parameter		PASC		
	Overall (*N* = 319)	No (*N* = 176)	Yes (*N* = 143)	*p*-Value
Country				0.001
Austria	14 (4.4%)	6 (3.4%)	8 (5.6%)	-
Germany	82 (26%)	34 (19%)	48 (34%)	-
Spain	22 (6.9%)	16 (9.1%)	6 (4.2%)	-
France	18 (5.6%)	6 (3.4%)	12 (8.4%)	-
North Macedonia	92 (29%)	64 (36%)	28 (20%)	-
Poland	76 (24%)	43 (24%)	33 (23%)	-
Sweden	15 (4.7%)	7 (4.0%)	8 (5.6%)	-
Age [years]	55 (41–65)	51 (38–63)	57 (47–68)	0.001
Ethnicity				-
Asian	1 (0.3%)	1 (0.6%)	0 (0%)	-
African	1 (0.3%)	0 (0%)	1 (0.7%)	-
Hispanic	3 (0.9%)	2 (1.1%)	1 (0.7%)	-
Other	5 (1.6%)	2 (1.1%)	3 (2.1%)	-
Unknown	20 (6.3%)	14 (8.0%)	6 (4.2%)	-
White	289 (91%)	157 (89%)	132 (92%)	-
Sex [1 = Male]	157 (49%)	89 (51%)	68 (48%)	0.592
BMI [kg/m^2^]	26.8 (24.7–30.3)	26.3 (24.2–30.1)	27.2 (25.4–30.7)	0.012
Smoking Status [1 = Yes]	41 (13%)	27 (15%)	14 (9.8%)	0.141
eGFR [mL/min/1.73 m^2^]	99 (88–114)	103 (90–116)	93 (84–110)	0.010
Systolic Blood Pressure [mmHg]	124 (115–135)	124 (115–133)	124 (115–137)	0.343
Diastolic Blood Pressure [mmHg]	77 (70–83)	77 (70–83)	77 (69–85)	0.775
WHO				<0.001
1	115 (36%)	87 (49%)	28 (20%)	-
2	10 (3.1%)	4 (2.3%)	6 (4.2%)	-
3	46 (14%)	17 (9.6%)	29 (20%)	-
4	92 (29%)	43 (24%)	49 (34%)	-
5	40 (13%)	20 (11%)	20 (14%)	-
6	16 (5.0%)	5 (2.8%)	11 (7.7%)	-
Patient Status at Inclusion [1 = Hospitalized]	194 (61%)	85 (48%)	109 (76%)	<0.001
Presence of Diabetes [1 = Yes]	53 (17%)	20 (11%)	33 (23%)	0.005
History of Hypertension [1 = Yes]	124 (39%)	53 (30%)	71 (50%)	0.001
History of Heart Failure [1 = Yes]	16 (5.0%)	5 (2.8%)	11 (7.7%)	0.048
History of Heart Disease [1 = Yes]	23 (7.2%)	6 (3.4%)	17 (11.9%)	0.004
History of Cancer [1 = Yes]	15 (4.7%)	7 (4.0%)	8 (5.6%)	0.532
Duration of Illness [months]	9 (2–9)	1 (0–1)	9 (4–9)	<0.001
Number of Work Days Missed [days]	30 (14–90)	21 (14–45)	53 (23–120)	0.001

Legend: *N*—number of patients; PASC—post-acute sequelae of SARS-CoV-2. The “WHO” parameter refers to the scores according to the WHO 8-point clinical progression scale. Values are shown as *N* (%) or median (IQR25–IQR75).

**Table 3:** Characteristics of ambulatory and hospitalized patients.

Parameter		Hospitalized		
	Overall (*N* = 319)	No (*N* = 125)	Yes (*N* = 194)	*p*-Value
Country				<0.001
Austria	14 (4.4%)	0 (0%)	14 (7.2%)	-
Germany	82 (26%)	4 (3.2%)	78 (40%)	-
Spain	22 (6.9%)	0 (0%)	22 (11%)	-
France	18 (5.6%)	0 (0%)	18 (9.3%)	-
North Macedonia	92 (29%)	80 (64%)	12 (6.2%)	-
Poland	76 (24%)	41 (33%)	35 (18%)	-
Sweden	15 (4.7%)	0 (0%)	15 (7.7%)	-
PASC [1 = Yes]	143 (45%)	34 (27%)	109 (56%)	<0.001
Age [years]	55 (41–65)	43 (34–54)	61 (51–71)	<0.001
Ethnicity				-
Asian	1 (0.3%)	0 (0%)	1 (0.5%)	-
African	1 (0.3%)	0 (0%)	1 (0.5%)	-
Hispanic	3 (0.9%)	0 (0%)	3 (1.5%)	-
Other	5 (1.6%)	0 (0%)	5 (2.6%)	-
Unknown	20 (6.3%)	0 (0%)	20 (10%)	-
White	289 (91%)	125 (100%)	164 (85%)	-
Sex [1 = Male]	157 (49%)	49 (39%)	108 (56%)	0.004
BMI [kg/m^2^]	26.8 (24.7–30.3)	26.5 (23.3–30.7)	26.9 (24.9–30.1)	0.185
Smoking Status [1 = Yes]	41 (13%)	34 (27%)	7 (3.6%)	<0.001
eGFR [mL/min/1.73 m^2^]	99 (88–114)	107 (98–117)	90 (76–109)	<0.001
Systolic Blood Pressure [mmHg]	124 (115–135)	123 (112–128)	126 (117–138)	<0.001
Diastolic Blood Pressure [mmHg]	77 (70–83)	78 (70–81)	76 (69–84)	0.581
WHO				<0.001
1	115 (36%)	115 (92%)	0 (0%)	-
2	10 (3.1%)	10 (8.0%)	0 (0%)	-
3	46 (14%)	0 (0%)	46 (24%)	-
4	92 (29%)	0 (0%)	92 (47%)	-
5	40 (13%)	0 (0%)	40 (21%)	-
6	16 (5.0%)	0 (0%)	16 (8.2%)	-
Presence of Diabetes [1 = Yes]	53 (17%)	3 (2.4%)	50 (26%)	<0.001
History of Hypertension [1 = Yes]	124 (39%)	24 (19%)	100 (52%)	<0.001
History of Heart Failure [1 = Yes]	16 (5.0%)	1 (0.8%)	15 (7.7%)	0.006
History of Heart Disease [1 = Yes]	23 (7.2%)	1 (0.8%)	22 (11.3%)	<0.001
History of Cancer [1 = Yes]	15 (4.7%)	1 (0.8%)	14 (7.2%)	0.021
Duration of Illness [months]	9 (2–9)	9 (2–9)	9 (3–9)	0.446
Number of Work Days Missed [days]	30 (14–90)	20 (14–29)	45 (21–120)	<0.001

Legend: *N*—number of patients. The “WHO” parameter refers to the scores according to the WHO 8-point clinical progression scale. Values are shown as *N* (%) or median (IQR25–IQR75).

**Table 4:** Characteristics of the groups divided by age.

Parameter		Age Group					
	Overall (*N* = 319)	20–39 Years (*N* = 69)	40–49 Years (*N* = 63)	50–58 Years (*N* = 61)	59–68 Years (*N* = 67)	69–90 Years (*N* = 59)	*p*-Value
Country							-
Austria	14 (4.4%)	2 (2.9%)	5 (7.9%)	4 (6.6%)	1 (1.5%)	2 (3.4%)	-
Germany	82 (26%)	7 (10%)	6 (9.5%)	17 (28%)	19 (28%)	33 (56%)	-
Spain	22 (6.9%)	4 (5.8%)	3 (4.8%)	4 (6.6%)	7 (10%)	4 (6.8%)	-
France	18 (5.6%)	1 (1.4%)	4 (6.3%)	2 (3.3%)	6 (9.0%)	5 (8.5%)	-
North Macedonia	92 (29%)	37 (54%)	27 (43%)	16 (26%)	10 (15%)	2 (3.4%)	-
Poland	76 (24%)	17 (25%)	16 (25%)	15 (25%)	18 (27%)	10 (17%)	-
Sweden	15 (4.7%)	1 (1.4%)	2 (3.2%)	3 (4.9%)	6 (9.0%)	3 (5.1%)	-
PASC [1 = Yes]	143 (45%)	19 (28%)	27 (43%)	32 (52%)	34 (51%)	31 (53%)	0.016
Ethnicity							-
Asian	1 (0.3%)	0 (0%)	0 (0%)	0 (0%)	1 (1.5%)	0 (0%)	-
African	1 (0.3%)	0 (0%)	0 (0%)	1 (1.6%)	0 (0%)	0 (0%)	-
Hispanic	3 (0.9%)	2 (2.9%)	0 (0%)	0 (0%)	1 (1.5%)	0 (0%)	-
Other	5 (1.6%)	1 (1.4%)	3 (4.8%)	1 (1.6%)	0 (0%)	0 (0%)	-
Unknown	20 (6.3%)	2 (2.9%)	3 (4.8%)	4 (6.6%)	7 (10%)	4 (6.8%)	-
White	289 (91%)	64 (93%)	57 (90%)	55 (90%)	58 (87%)	55 (93%)	-
Sex [1 = Male]	157 (49%)	36 (52%)	28 (44%)	28 (46%)	35 (52%)	30 (51%)	0.846
BMI [kg/m^2^]	26.8 (24.7–30.3)	26.1 (22.9–30.3)	26.9 (24.1–30.9)	27.2 (25.1–33.1)	27.8 (25.8–30.5)	26.5 (24.5–28.3)	0.025
Smoking Status [1 = Yes]	41 (13%)	17 (25%)	14 (22%)	7 (11%)	3 (4.5%)	0 (0%)	<0.001
eGFR [mL/min/ 1.73 m^2^]	99 (88–114)	118 (113–122)	105 (100–110)	96 (92–101)	90 (83–100)	77 (62–91)	<0.001
Systolic Blood Pressure [mmHg]	124 (115–135)	120 (113–125)	124 (113–130)	126 (115–136)	127 (118–141)	126 (115–140)	0.004
Diastolic Blood Pressure [mmHg]	77 (70–83)	77 (70–81)	75 (69–85)	80 (72–84)	76 (70–85)	75 (69–83)	0.377
WHO							-
1	115 (36%)	46 (67%)	34 (54%)	21 (34%)	14 (21%)	0 (0%)	-
2	10 (3.1%)	6 (8.7%)	1 (1.6%)	3 (4.9%)	0 (0%)	0 (0%)	-
3	46 (14%)	7 (10%)	9 (14%)	10 (16%)	11 (16%)	9 (15%)	-
4	92 (29%)	5 (7.2%)	9 (14%)	19 (31%)	24 (36%)	35 (59%)	-
5	40 (13%)	5 (7.2%)	6 (9.5%)	6 (9.8%)	12 (18%)	11 (19%)	-
6	16 (5.0%)	0 (0%)	4 (6.3%)	2 (3.3%)	6 (9.0%)	4 (6.8%)	-
Patient Status at Inclusion [1 = Hospitalized]	194 (61%)	17 (25%)	28 (44%)	37 (61%)	53 (79%)	59 (100%)	<0.001
Presence of Diabetes [1 = Yes]	53 (17%)	1 (1.4%)	3 (4.8%)	9 (15%)	18 (27%)	22 (37%)	<0.001
History of Hypertension [1 = Yes]	124 (39%)	5 (7.2%)	13 (21%)	24 (39%)	41 (61%)	43 (73%)	<0.001
History of Heart Failure [1 = Yes]	16 (5.0%)	0 (0%)	0 (0%)	2 (3.3%)	4 (6.0%)	10 (17%)	<0.001
History of Heart Disease [1 = Yes]	23 (7.2%)	0 (0%)	0 (0%)	2 (3.3%)	6 (9.0%)	15 (25%)	<0.001
History of Cancer [1 = Yes]	15 (4.7%)	0 (0%)	1 (1.6%)	5 (8.2%)	5 (7.5%)	4 (6.8%)	0.152
Duration of Illness [months]	9 (2–9)	4 (1–9)	9 (2–9)	4 (2–9)	9 (4–9)	9 (6.5–9)	0.030
Number of Work Days Missed [days]	30 (14–90)	17 (14–29)	30 (14–60)	35 (20–101)	60 (28–101)	100 (0–200)	0.017

Legend: *N*—number of patients. The “WHO” parameter refers to the scores according to the WHO 8-point clinical progression scale. Values are shown as *N* (%) or median (IQR25–IQR75).

**Table 5:** Comparison of the frequency of consulting specialists among ambulatory and hospitalized patients.

Parameter	Ambulatory	Hospitalized	*p*-Value
Pneumologist [Yes/No]	Yes: 2 No: 10	Yes: 56 No: 43	0.057
Cardiologist [Yes/No]	Yes: 4 No: 8	Yes: 39 No: 57	0.609
Neurologist [Yes/No]	Yes: 1 No: 11	Yes: 18 No: 77	0.391
Psychiatrist [Yes/No]	Yes: 0 No: 12	Yes: 7 No: 89	0.395
Physiotherapist [Yes/No]	Yes: 0 No: 12	Yes: 27 No: 69	0.065
General practitioner [Yes/No]	Yes: 10 No: 2	Yes: 76 No: 26	0.751

**Table 6:** Comparison of the frequency of consulting specialists among non-PASC and PASC patients.

Parameter	Non-PASC	PASC	*p*-Value
Pneumologist [Yes/No]	Yes: 12 No: 19	Yes: 46 No: 34	0.308
Cardiologist [Yes/No]	Yes: 6 No: 25	Yes: 37 No: 40	0.017
Neurologist [Yes/No]	Yes: 3 No: 27	Yes: 16 No: 61	0.415
Psychiatrist [Yes/No]	Yes: 1 No: 29	Yes: 6 No: 72	0.712
Physiotherapist [Yes/No]	Yes: 4 No: 26	Yes: 23 No: 55	0.214
General practitioner [Yes/No]	Yes: 23 No: 9	Yes: 63 No: 19	0.706

**Table 7:** Characteristics of the groups divided by anxiety and depression levels.

Parameter	No Anxiety/ Depression	Slight Anxiety/ Depression	Moderate Anxiety/ Depression	Severe Anxiety/ Depression	Extreme Anxiety/ Depression	***p*-Value**
Sex [W/M]	W: 62 M: 69	W: 27 M: 15	W: 8 M: 17	W: 5 M: 3	W: 4 M: 1	0.067
PASC [Yes/No]	Yes: 53 No: 78	Yes: 23 No: 19	Yes: 22 No: 3	Yes: 7 No: 1	Yes: 4 No: 1	<0.001
Patient Status at Inclusion [H/A]	H: 96 A: 35	H: 37 A: 5	H: 24 A: 1	H: 7 A: 1	H: 5 A: 0	0.046

Legend: *N*—number of patients; W—women; M—men; PASC—post-acute sequelae of SARS-CoV-2; H—hospitalized; A—ambulatory.

**Table 8:** The relationship between the selected parameters and the risk of developing PASC.

Variables	Category	Non-PASC	PASC	*p*-Value	OR	95% CI
Country	Poland	43	33	-	1.000	-
	Austria	6	8	0.577	0.688	0.184–2.567
	Germany	34	48	0.685	0.849	0.386–1.870
	Spain	16	6	0.042	0.298	0.093–0.959
	France	6	12	0.805	1.191	0.298–4.764
	North Macedonia	64	28	0.726	0.874	0.412–1.853
	Sweden	7	8	0.601	0.720	0.210–2.470
Age	-	-	-	0.949	0.999	0.978–1.021
Sex	Men	89	68	-	1.000	-
	Women	87	75	0.150	1.469	0.870–2.480
BMI	-	-	-	0.0854	1.045	0.994–1.098
eGFR	-	-	-	0.457	1.003	0.994–1.012
Systolic Blood Pressure	-	-	-	0.474	0.994	0.978–1.011
Smoking Status	No	149	129	-	1.000	-
	Yes	27	14	0.498	1.313	0.597–2.884
Status at Inclusion	Ambulatory	91	34	-	1.000	-
	Hospitalized	85	109	0.001	4.003	1.728–9.276
Presence of Diabetes	No	156	110	-	1.000	-
	Yes	20	33	0.811	1.094	0.524–2.283
History of Hypertension	No	123	72	-	1.000	-
	Yes	53	71	0.260	1.409	0.776–2.559
History of Heart Failure	No	171	132	-	1.000	-
	Yes	5	11	0.369	0.326	0.028–3.767
History of Heart Disease	No	170	126	-	1.000	-
	Yes	6	17	0.115	5.902	0.649–53.668

Legend: PASC—post-acute sequelae of SARS-CoV-2; OR—odds ratio; 95% CI—95% confidence intervals.

## Data Availability

The data presented in this study are available on request from the corresponding author. The data are not publicly available due to a lack of patients’ consent to make their data public.

## Supplementary Materials

The following supporting information can be downloaded at https://www.mdpi.com/article/10.3390/jcm15103681/s1, Questionnaire S1: Long-/Post-COVID-19 Questionnaire; Figure S1. (A)–(H): Functional independence and rehabilitation use before and after COVID-19: Wroclaw vs. other patients; Figure S2. (A)–(H): Mental health and cognitive symptoms before and after COVID-19: Wroclaw vs. other patients; Figure S3. (A)–(H): Olfactory, gustatory, and thrombotic complications after COVID-19: Wroclaw vs. other patients; Figure S4. (A)–(D): COVID-19 recurrence and outpatient follow-up: Wroclaw vs. other patients.

## Abbreviations

The following abbreviations are used in this manuscript:

PASC	Post-acute sequelae of COVID-19
COVID-19	Coronavirus disease 2019
SARS-CoV-2	Severe acute respiratory syndrome coronavirus 2
CDC	Centers for Disease Control and Prevention
UriCoV	“URInary Peptidomic Patterns of Long-COVID Syndrome” study
CRIT-COV-U	“Prospective Validation of a Proteomic Urine Test for Early and Accurate Prognosis of Critical Course Complications in Patients with SARS-CoV-2 Infection” study
WHO	World Health Organization
eGFR	Estimated glomerular filtration rate
OR	Odds ratio
CI	Confidence interval
POTS	Postural orthostatic tachycardia syndrome
PICS	Post-intensive care syndrome
PTSD	Post-traumatic stress disorder
DLCO	Diffusing capacity for carbon monoxide
ME/CFS	Myalgic encephalomyelitis/chronic fatigue syndrome
