# Supplementary material for: Post-Acute Sequelae of COVID-19 (PASC) in Hospitalized and Ambulatory Patients: A Comparative Study
Source: J Clin Med. 2026 May 11;15(10):3681. doi: 10.3390/jcm15103681 (PMC13207122; doi:10.3390/jcm15103681)
Supplement: Supplementary file 1 [file jcm-15-03681-s001.zip › jcm-4228026-supplementary.pdf]

Supplementary materials

Questionnaire S1. Long-/Post-COVID-19 Questionnaire.

Long-/Post-COVID-19 Questionnaire

First name, last name: ____

Date of birth: ______. ______. ______________ (DD.MM.YYYY)

When answering this questionnaire, please think back to your first COVID-19 illness.

Did any symptoms/complaints persist since your first acute COVID-19 illness?	Yes<br>No	
Did any new complaints arise after your first acute COVID-19 illness (within the following 3 months after the acute illness)?	Yes<br>No	
Did you have any symptoms/complaints that had become a new health limitation since your first acute COVID-19 illness?	Yes<br>No	
Did a pre-existing underlying condition worsen since your first acute COVID-19 illness?	Yes<br>No	
**If YES was checked for at least 1 of the 4 previous questions:**		
How long did these complaints last after your acute COVID-19 illness?	< 4 weeks<br>4 bis < 8 weeks<br>2 bis < 6 months<br>6 bis < 12 months<br>12 bis < 24 months<br>Persisting until today	

**Please think back to the time right <u>after</u> your first acute COVID-19 illness.**		
Could you live alone without any assistance from another person?<br>(e.g. independently being able to eat, walk, use the toilet and manage routine daily hygiene)	Yes<br>No	
Were there duties/activities at home or at work which you were/are no longer able to perform yourself?	Yes<br>No	
Did you suffer from symptoms, pain, depression, or anxiety?	Yes<br>No	
Did you need to avoid or reduce duties/activities or spread these over time?	Yes<br>No	
**If NO was checked for question 1 or YES was checked for at least 1 of the following 3 questions:**		
Did this limitation in your independence and ability to perform occur <u>after</u> your acute COVID-19 illness?	Yes<br>No	

**How much was your daily life affected in the period following your first acute COVID-19 illness?**<br><br>**Please indicate which one of the following statements applies to you most.**<br>**Please tick only 1 out of the 5 boxes.**	
I had no limitations in my everyday life and no symptoms, pain, depression, or anxiety.	
I had negligible limitations in my everyday life as I could perform all usual duties/activities, although I still had persistent symptoms, pain, depression, or anxiety.	
I suffered from limitations in my everyday life as I occasionally had to avoid or reduce usual duties/activities or had to spread these over time due to symptoms, pain, depression, or anxiety. I was, however, able to perform all activities without any assistance.	
I suffered from limitations in my everyday life as I was not able to perform all usual duties/activities due to symptoms, pain, depression, or anxiety. I was, however, able to take care of myself without any assistance.	
I suffered from severe limitations in my everyday life: I was not able to take care of myself and therefore I was dependent on nursing and/or assistance from another person due to symptoms, pain, depression, or anxiety.	

**Please think back to the time right <u>after</u> your first acute COVID-19 illness.**<br><br>**Please tick YES only if the symptom occurred or increased during this period compared to the condition before your COVID-19 illness.**		
Were you more breathless than <u>before</u> your acute COVID-19 illness?	Yes<br>No	
Did you have more cough or a different kind of cough compared to <u>before</u> your acute COVID-19 illness? (different from any cough you may have had before COVID-19)?	Yes<br>No	
Did you feel fatigued (worn out/lacking energy or zest) compared with how you were feeling before your COVID-19 illness?	Yes<br>No	
How was your physical strength? Did you feel so weak that it was limiting what you could do (more than <u>before</u> your COVID-19 illness?)	Yes<br>No	
Did you feel any aching in your muscles (myalgia)?	Yes<br>No	
Did you suffer from sleep disturbances? (new or increased occurrence compared to before your acute COVID-19 illness)	Yes<br>No	
Did you suffer from any nightmares or flashbacks (= occurrence of sudden, involuntary memories accompanied by strong emotions)?<br>(new or increased occurrence compared to <u>before</u> your acute COVID-19 illness)	Yes<br>No	
Was your mood low or did you feel down in the dumps/lacking in motivation/no pleasure in anything?<br>(new or increased occurrence compared to <u>before</u> your acute COVID-19 illness)	Yes<br>No	
Did you find yourself feeling anxious/worrying more than you used to <u>before</u> your acute COVID-19 illness?	Yes<br>No	
Did you have problems with your memory, concentration or with organising your thoughts, that started <u>after</u> your acute COVID-19 illness?	Yes<br>No	
Did you lose your sense of smell (anosmia)?	Yes<br>No	
Did you lose your sense of taste?	Yes<br>No	

Did you lose more than 3 kg of weight in the 3 following months after your COVID-19 illness?	Yes<br>No	
Did you suffer from any other complaints (not listed here), that arose after your acute COVID-19 illness or have persist since then?	Yes<br>No	
**If the previous question was answered with YES:**<br><br>Please name your complaints!		

Have you contracted COVID-19 again since your discharge from the hospital/after your first acute illness?	Yes<br>No	
Did you see an outpatient doctor due to persistent or new complaints or worsening of an underlying condition after your first acute COVID-19 illness?	Yes<br>No	
Were you readmitted as an inpatient due to persistent or new complaints or worsening of an underlying condition after your first acute COVID-19 illness?	Yes<br>No	
Was a rehabilitation measure (e.g. rehab, physiotherapy, occupational therapy) carried out following your first acute COVID-19 illness?	Yes<br>No	
Have you suffered a stroke, pulmonary embolism (blood clot in a blood vessel of the lung) or thrombosis (blood clot in a vein) since your discharge from the hospital/your first COVID-19 illness?	Yes<br>No	
Were you employed before your first acute COVID-19 illness started?	Yes<br>No	
**If YES was checked in the previous question:**<br><br>How many days did you miss work due to persistent or new complaints or worsening of an underlying condition after your acute COVID-19 illness?	Days	
How many times were you vaccinated against SARS-CoV-2 (COVID-19) before you became ill (with COVID-19)?		
How many times in total have you been vaccinated against SARS-CoV-2 (COVID-19) so far?		

Would you be willing to participate in a telephone interview conducted by medical personnel as part of this study?	Yes<br>No	

Thank you very much for your participation!

The following are the symptoms and medical events associated with patients from the study population who were recruited in Poland. The division is based on PASC (long COVID) diagnosis and place of residence (patients from Wroclaw are presented separately).

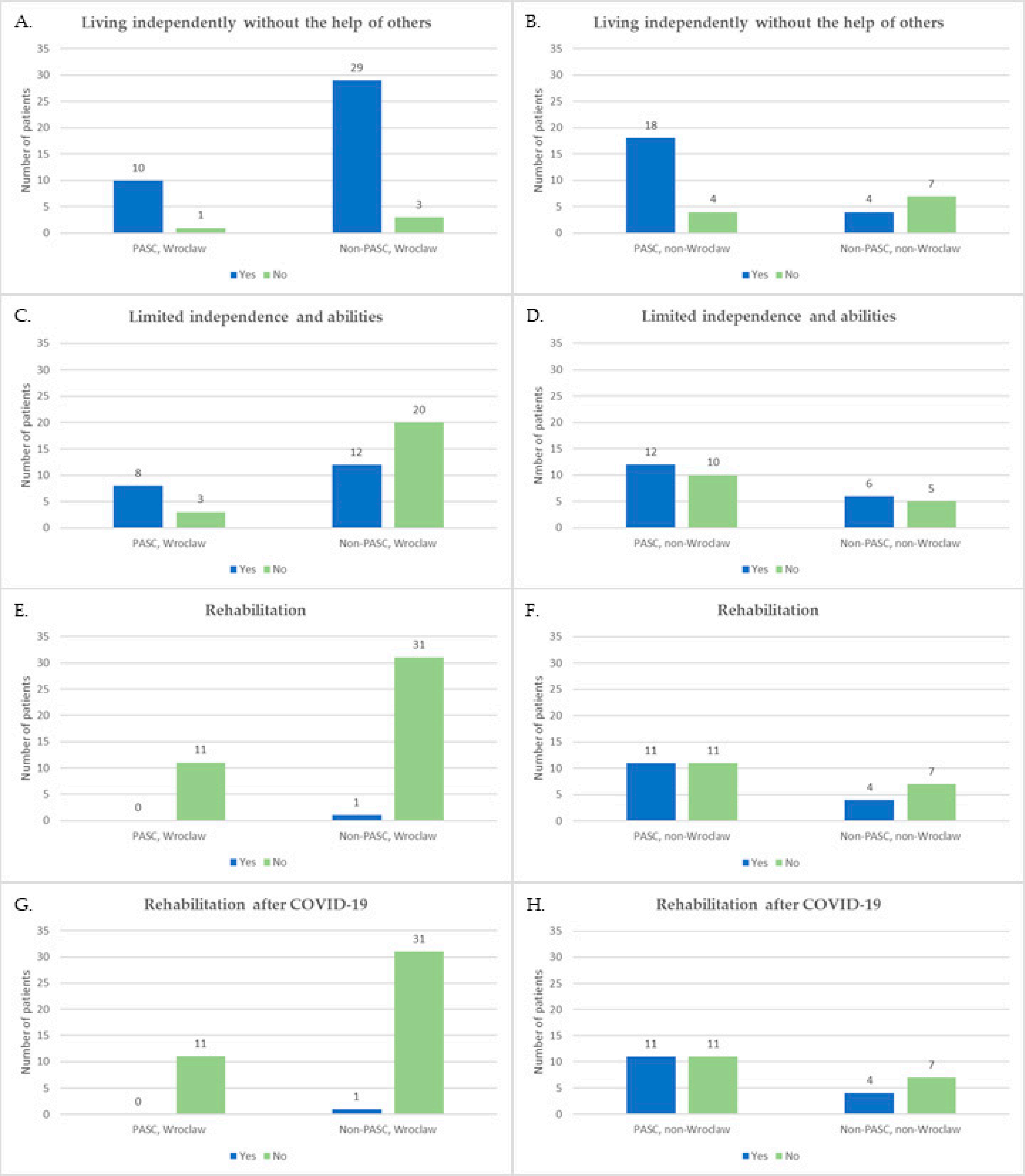

Figure S1. (A)–(H): Functional independence and rehabilitation use before and after COVID-19: Wroclaw vs other patients. **(A)–(B):** Ability to live independently without assistance. **(C)–(D):** Self-reported limitations in independence. **(E)–(F):** Use of rehabilitation services. **(G)–(H):** Use of rehabilitation after COVID-19. **(A, C, E, G: patients from Wroclaw; B, D, F, H: other patients)**

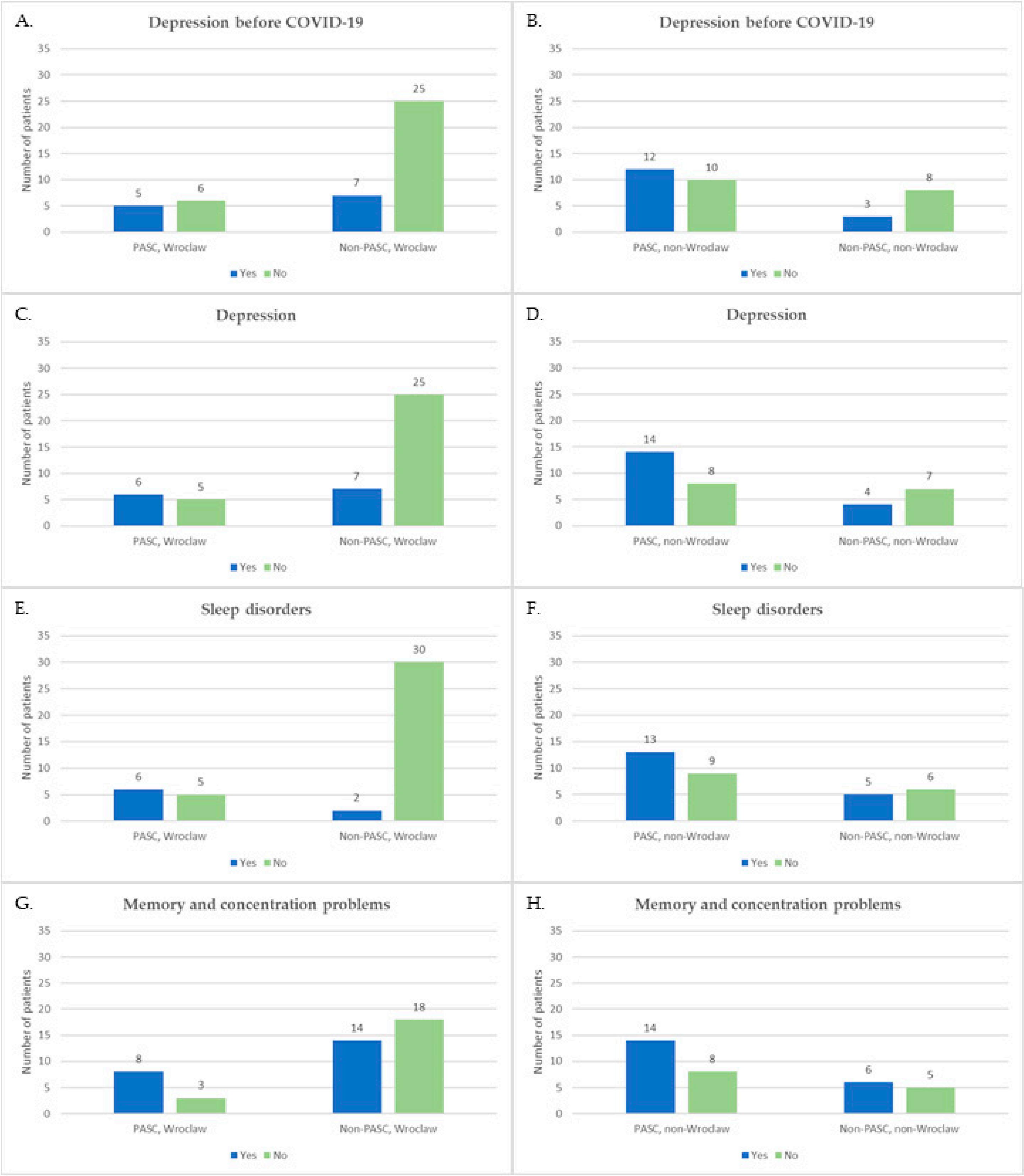

Figure S2. (A)–(H): Mental health and cognitive symptoms before and after COVID-19: Wroclaw vs other patients.
(A)–(B): Prevalence of depression before COVID-19. **(C)–(D):** Prevalence of depression. **(E)–(F):** Prevalence of sleep disorders. **(G)–(H):** Memory and concentration problems. **(A, C, E, G: patients from Wroclaw; B, D, F, H: other patients)**

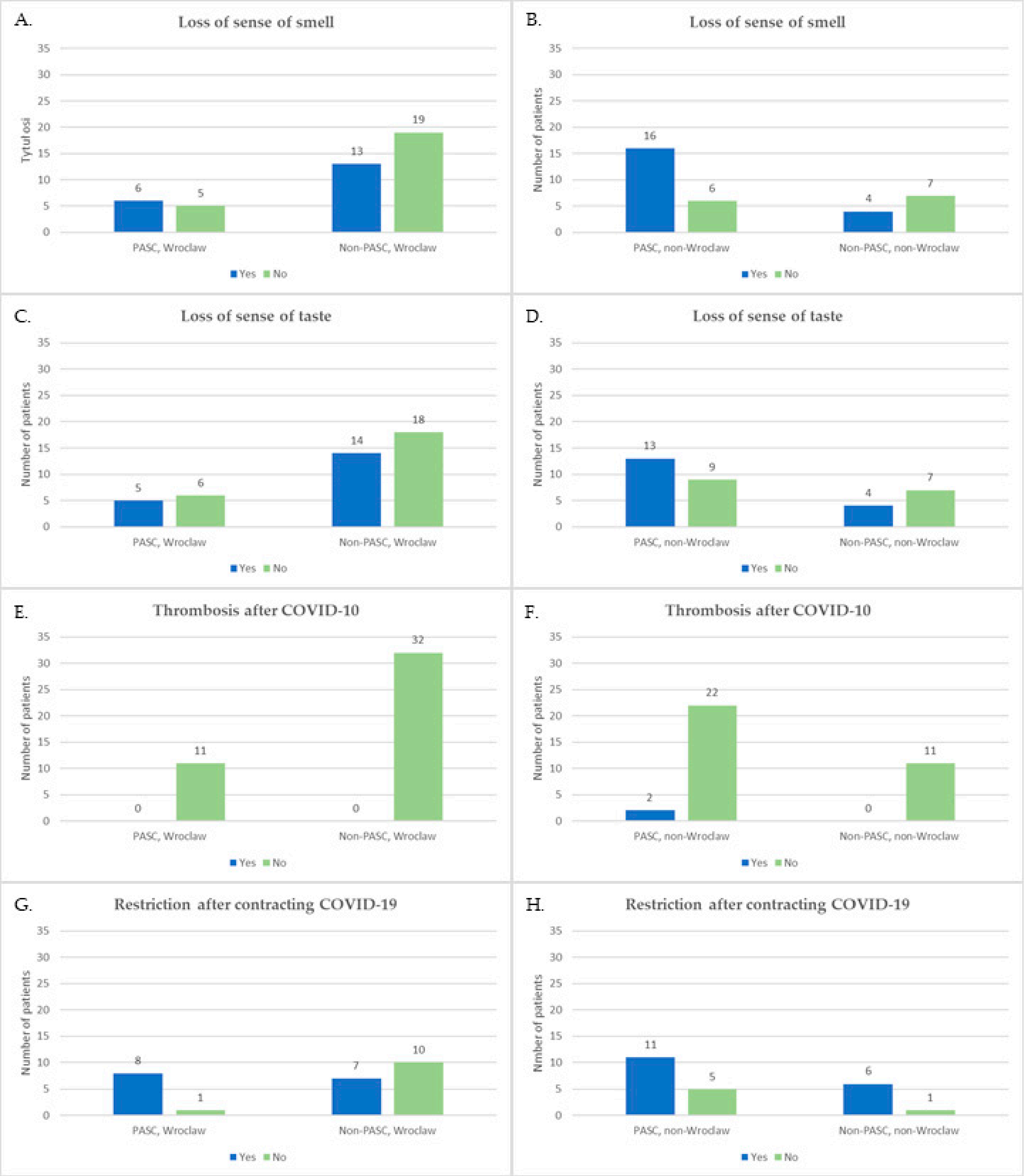

Figure S3. (A)–(H): Olfactory, gustatory and thrombotic complications after COVID-19: Wroclaw vs other patients.
(A)–B): Loss of sense of smell. **(C)–(D):** Loss of sense of taste. **(E)–(F):** Thrombosis after COVID-19. **(G)–(H):** Post-COVID functional restrictions. **(A, C, E, G: patients from Wroclaw; B, D, F, H: other patients)**

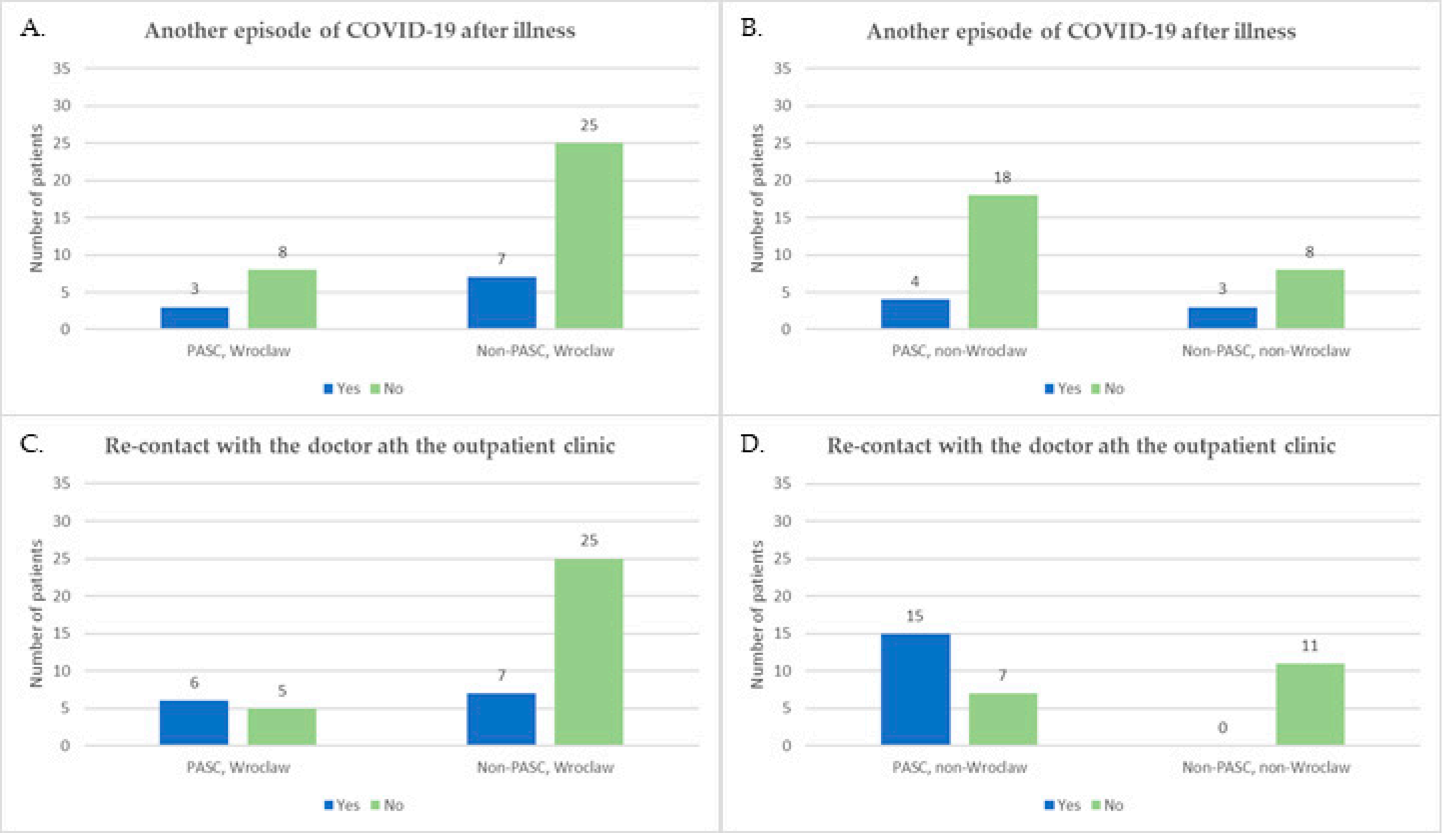

Figure S4. (A)–(H): COVID-19 recurrence and outpatient follow-up: Wroclaw vs other patients.
(A)–(B): Occurrence of a subsequent episode of COVID-19 after the initial illness. **(C)–(D):** Re-contact with a physician at an outpatient clinic. **(A, C: patients from Wroclaw; B, D: other patients)**
